# Pleural Effusion or Main Left Bronchus Mucus Obstruction: To Drain or Not to Drain? Decision-Making for Young Surgeon on Call

**DOI:** 10.1155/2018/3180575

**Published:** 2018-01-28

**Authors:** Danilo Coco, Silvana Leanza

**Affiliations:** ^1^Madre Teresa of Calcutta Hospital, Schiavonia, Padova, Italy; ^2^Carlo Urbani Hospital, Jesi, Ancona, Italy

## Abstract

Mucous plugs occur in a number of pulmonary conditions. Central right or left bronchus mucus plug causes complete pulmonary collapse making it an emergency life-threatening case. We describe the case of an 80-year-old man that, in postoperative period after a urological intervention, has had a progressive tachypnea and dyspnea during hospitalization for urological problems. Young surgeon on call was called.

## 1. Introduction

Mucous plugs occur in a number of pulmonary conditions such as bronchial asthma, pulmonitis, cystic fibrosis, and bronchiectasis and in various types of obstructive pathology [1]. In the elderly, it can appear for restless situations such as long postoperative period of bed rest. It is difficult to recognize and has differential diagnosis with pleural effusion or pneumothorax.

## 2. Case Report

**Figure 1:**
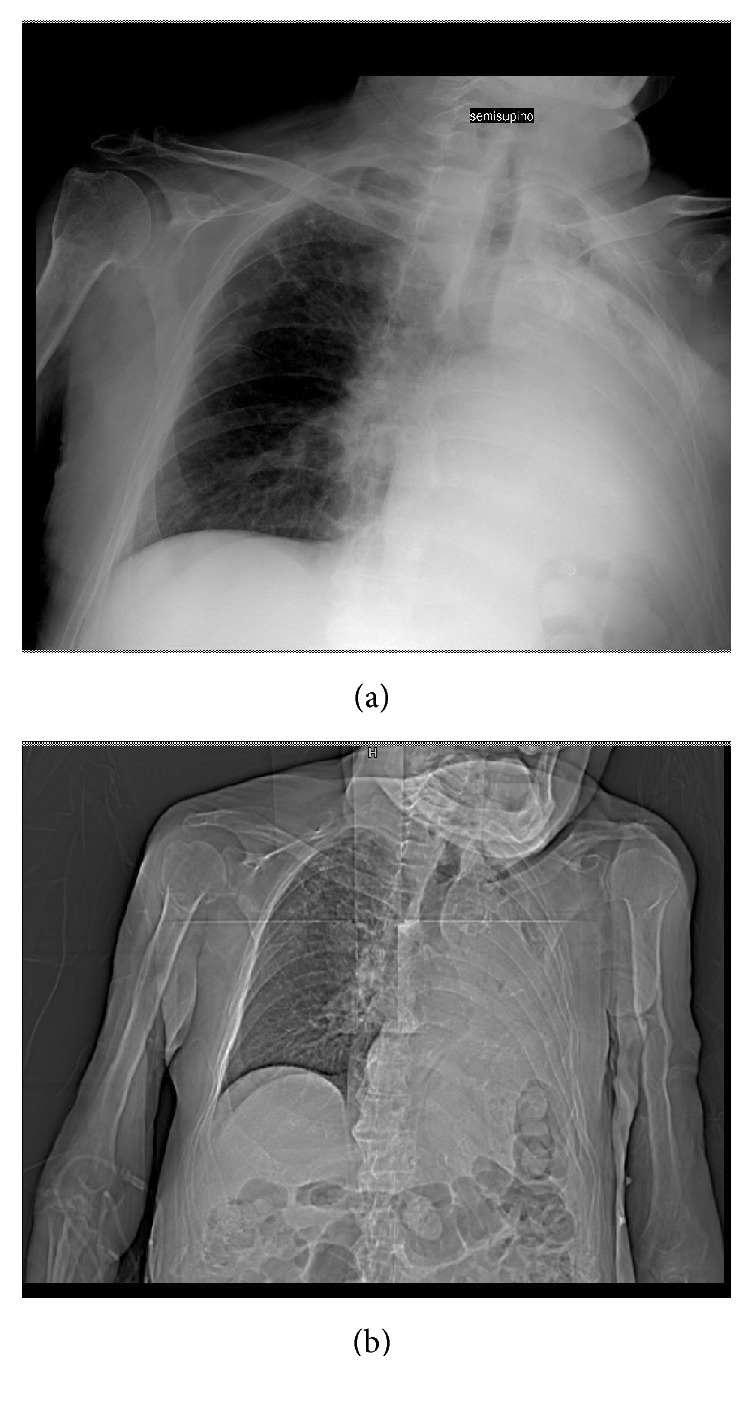
Complete left pulmonary opacification.

**Figure 2:**
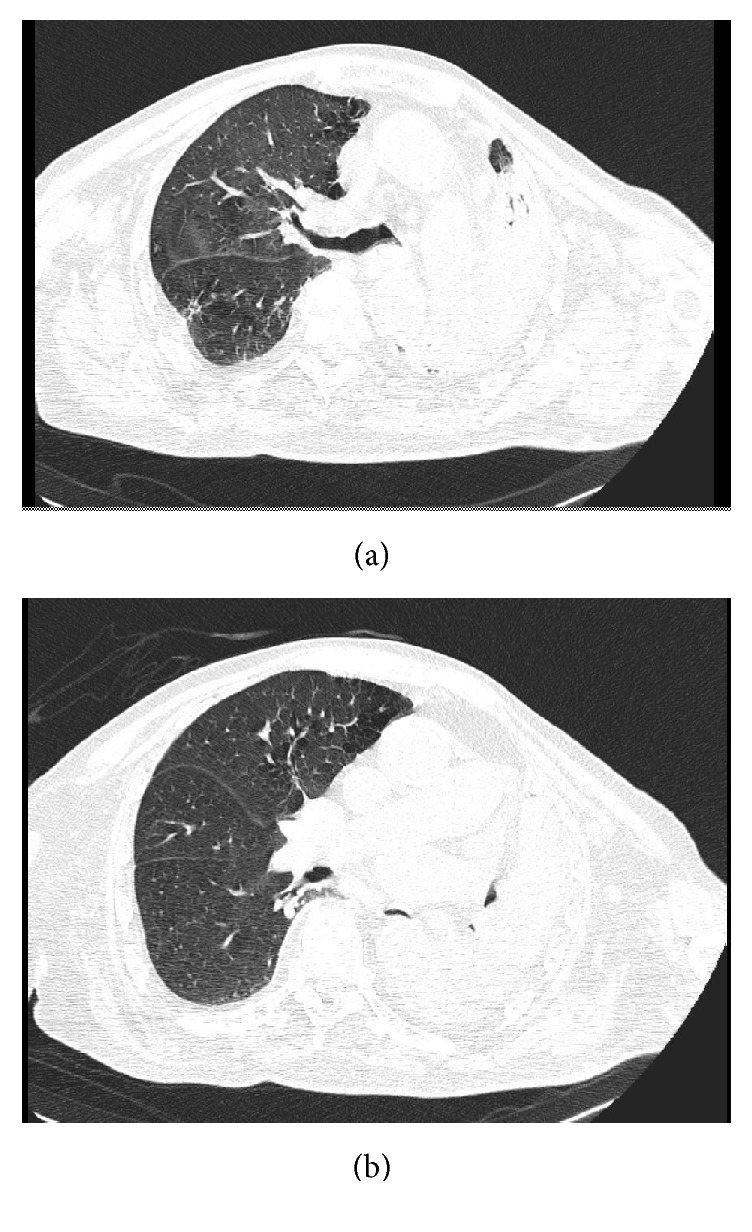
Chest CT scan: left bronchus mucus plug.

**Figure 3:**
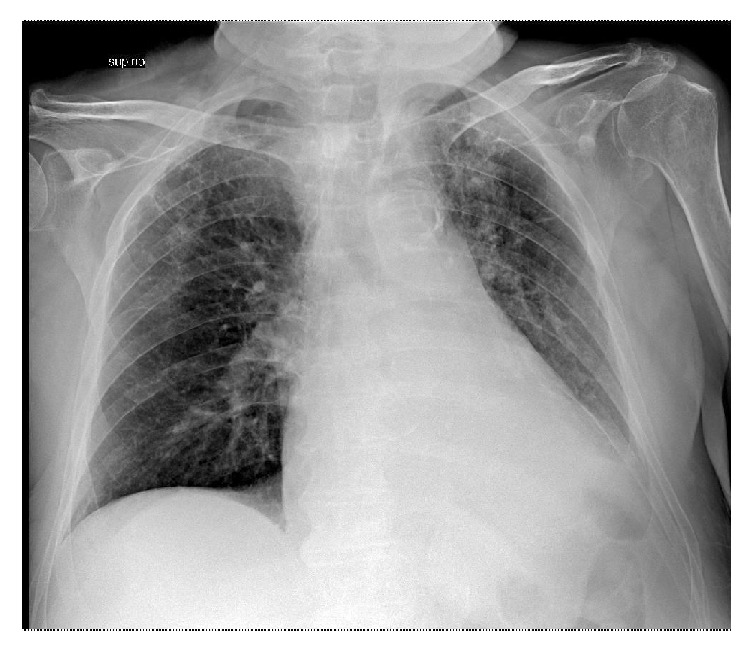
Chest radiography after bronchoscopy.

**Figure 4:**
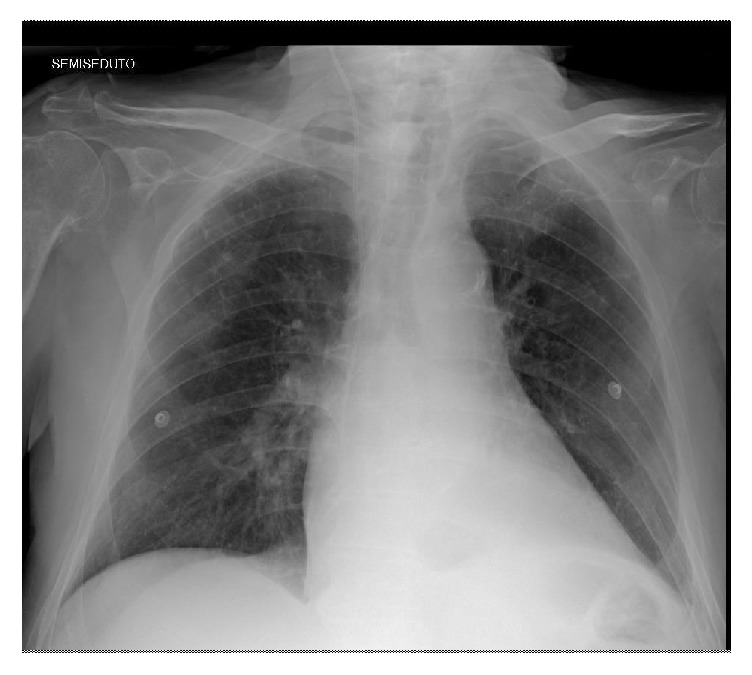
Chest radiography two months later.

An 80-year-old man hospitalized in urologic department for BPH with history of hypertension, diabetes mellitus, and severe obstructive pulmonary pathology. The interdivisional surgeon of surgery department was called because the patient had increasing shortness of breath and a cough productive of small amounts of yellow sputum, low blood pressure, discolored skin or nails, confusion and extreme tiredness, muscle fatigue, and general weakness. Physical examination of chest demonstrated normal tracheal breath sound, asymmetric thoracic movement, absent breath sounds, absent left bronchovascular breath sound, and increased vocal resonance. Arterial blood gas (ABG) demonstrated respiratory acidosis. A chest radiography showed opacification of and volume loss in the left lung (Figures 1(a) and 1(b)). It was difficult to differentiate massive pleural effusion. A thoracic CT scan confirmed the complete lung atelectasis without pleural effusion (Figures 2(a) and 2(b)). The initial choice of chest tube placement was converted in a bronchoscopy. Bronchoscopy revealed a large mucous plug completely occluding the left main bronchus. It was necessary to remove the plug. The next X-ray showed left lung fully expanded after the plug was removed (Figure 3). The patient's breathing also improved substantially. One month later, chest radiography showed a complete resolution (Figure 4).

## 3. Pathogenesis

Mucus plug is an accumulation of desquamating mucus cells of bronchus and mucus that make an obstruction in the elderly and in all patients that have lost cough capacity. A sectorial atelectasis appears when the mucus plug occludes a peripheral bronchus. If it occludes the main bronchus a complete pulmonary collapse occurs.

## 4. Clinical Features

The most urgent clinical features are tachypnea, dyspnea, alteration pressure or frequency, and alteration of PO2 PCO2 in EGA; accessory respiratory muscles evidence; reduction of pulmonary sound being dull on percussion. Differential diagnosis is between pleural effusion or pulmonary massive atelectasis.

## 5. Diagnostic Evaluations 

Thoracic X-ray is the first diagnostic evaluation. It demonstrates complete pulmonary hypodiaphania. Thoracic CT scan is useful when the doubt exists. It has more sensibility and specificity to prove mucus plug. Bronchoscopy with flexible and rigid instruments is diagnostic and resolutive [2, 3].

## 6. Treatment 

Treatment with antibiotics, corticosteroids, hydration, and chest physiotherapy often produces improvement. In emergency situation it is the first choice. Bronchoscopy is required to achieve lung expansion [4, 5].

## 7. Conclusions 

Mucus plug in central pulmonary bronchus is a pathology that can occur in the elderly with restlessness associated with pulmonary or cardiologic pathology. For the surgeon it is important to distinguish it from pleural effusion to avoid chest tube drain.
